# Autonomy support in physical education and university students’ physical activity: indirect and conditional associations through motivation and environmental support

**DOI:** 10.3389/fpubh.2026.1854693

**Published:** 2026-05-20

**Authors:** Jiacheng Guo, Zongkui Zhou, Chenpeng Xu, Yun Li

**Affiliations:** 1 Central China Normal University, Wuhan, China; 2 California Baptist University, Riverside, CA, United States

**Keywords:** autonomy support, environmental support, motivation for physical activity, physical activity, physical education, university students

## Abstract

**Background:**

Insufficient physical activity among university students remains a significant public health concern. Although physical education (PE) provides an important context for promoting active lifestyles, previous research has focused mainly on motivation and behavioral outcomes within PE itself, leaving it unclear whether autonomy-supportive experiences in PE are associated with students’ out-of-class motivation for physical activity and their physical activity. In addition, the motivation–behavior link may vary across contextual conditions, highlighting the need to identify conditions associated with this process. Drawing on self-determination theory and the capability–opportunity–motivation–behavior model, this study examined whether motivation for physical activity may help explain the association between perceived autonomy support in PE and university students’ physical activity, and whether the association between motivation and physical activity varies by levels of environmental support.

**Methods:**

A cross-sectional online survey was conducted among 1,024 undergraduate students taking general PE courses at five universities across three cities in China. Participants completed measures of perceived autonomy support in PE, motivation for physical activity, environmental support for physical activity, physical activity, and physical literacy.

**Results:**

Perceived autonomy support in PE was positively associated with motivation for physical activity, which in turn was positively associated with physical activity. The direct association between autonomy support in PE and physical activity was not statistically supported after motivation for physical activity, environmental support, and their interaction were taken into account. Motivation may help explain the association between autonomy support in PE and physical activity, and environmental support was associated with a stronger link between motivation and physical activity. Accordingly, the indirect association between autonomy support in PE and physical activity via motivation was stronger under more supportive environmental conditions.

**Conclusion:**

These findings suggest that autonomy-supportive experiences in PE may be linked to students’ out-of-class motivation for physical activity and, indirectly, to their physical activity, while supportive environments may be associated with a stronger link between motivation and physical activity. Campus-based efforts to support physical activity among university students may therefore benefit from attention to autonomy-supportive teaching in PE and everyday environments in which motivation is more strongly associated with physical activity beyond the PE context.

## 1 Introduction

Globally, insufficient physical activity has become a prominent public health concern. Guthold et al. (1), based on a pooled analysis of data from 1.9 million participants across 168 countries, reported that the prevalence of insufficient physical activity among adults worldwide was 27.5% in 2016. Strain et al. (2) further showed, using data from 5.7 million participants across 163 countries and territories, that the global prevalence of insufficient physical activity among adults had risen to 31.3% in 2022, up from 23.4% in 2000 and 26.4% in 2010, suggesting that this global problem continues to worsen. As an important subgroup within the adult population, university students account for a substantial proportion of young adults in most developed countries (3). Entering university is often accompanied by lifestyle changes that may lead to weight gain, reduced physical activity, and increased sedentary behavior (4). Specifically, a meta-analysis found that university students reported an average of 7.29 h of sedentary time per day, which was higher than that of their non-university peers (3). Against this backdrop, insufficient physical activity is also prevalent among university students: previous reviews have suggested that approximately 40–50% of university students are insufficiently physically active (4), while a survey across 23 countries likewise found that 41.4% of university students did not meet recommended physical activity levels (5). However, longitudinal and intervention-based evidence suggests that regular physical activity may have protective effects on university students’ cardiovascular health, mental health, and well-being (6). Therefore, insufficient physical activity may represent an important risk factor for university students’ physical and mental health. Moreover, the university years are not only a critical stage in the educational trajectory, but also a key developmental period during which health behaviors and lifestyle patterns are established in early adulthood (3). Unhealthy behavioral habits formed during this period may persist into post-graduation adult life (7, 8). Taken together, it is particularly important to examine factors associated with physical activity among university students.

Sallis et al. (9) argued that the ecological model is an effective theoretical framework for understanding and promoting physical activity. This model emphasizes that, beyond individual-level determinants, physical activity should be understood as the product of multiple levels of influence, including interpersonal, organizational, physical environmental, and policy-related factors. In the university context, in addition to classroom instruction and educational settings (10), it is also necessary to consider the roles of interpersonal and social influences as well as environmental context and resources in shaping university students’ physical activity (11). From a public health and policy perspective, the costs of insufficient physical activity are reflected not only in damage to individual health but also in substantial societal and healthcare system burdens. Santos et al. (12) estimated that if global levels of insufficient physical activity remain unchanged, there will be 499.2 million new preventable cases of major non-communicable diseases by 2030, together with approximately INT$520 billion in direct healthcare costs. This highlights the importance of earlier attention to behavioral development and environmental support rather than responding passively after disease onset. At the same time, this issue is also aligned with the principles advocated in Sustainable Development Goals (SDGs) 3, 4, and 11, which emphasize promoting health and well-being, supporting holistic human development, and creating supportive environments (13). Therefore, examining environmental correlates of physical activity among university students is particularly important. Such research can help identify modifiable factors from a public health perspective and provide evidence to inform campus health promotion policies and university management practices. However, although prior research has highlighted the importance of educational, motivational, and environmental influences on physical activity, these factors have more often been examined separately than within an integrated framework in university populations. In particular, it remains insufficiently clear whether autonomy-supportive experiences in physical education are associated with university students’ out-of-class motivation for physical activity, and whether supportive environments help explain when motivation is more strongly associated with actual physical activity behavior.

Within the university context, students’ physical activity may be associated not only with individual factors but also with multiple layers of contextual support, including educational settings, social support, and environmental resources. School-based physical education is an important educational context associated with students’ physical activity (10), and physical education teachers, as one of the key facilitators of university students’ physical activity, may be linked to students’ sustained engagement in physical activity through autonomy-supportive teaching practices (14). However, although many studies have examined the relationship between autonomy support in physical education and physical activity, these studies have primarily focused on secondary school settings, while research investigating the association between autonomy support in physical education and university students’ physical activity remains relatively recent and limited (15). Moreover, although previous studies have examined the associations of autonomy support in physical education with motivation in physical education classes (16) and with participation in physical education classes (15), research examining whether autonomy support in university physical education is associated with out-of-class physical activity motivation and actual physical activity beyond the classroom context remains relatively limited. In addition, current models tend to explain university students’ physical activity intentions and motivation better than they explain actual physical activity behavior, highlighting the need for a more integrative perspective (17). Few studies have integrated educational contextual support, motivational processes, and environmental opportunity conditions within a single framework to examine when motivation for physical activity is more strongly associated with actual physical activity behavior. On this basis, the present study seeks to integrate self-determination theory (SDT) and the capability–opportunity–motivation–behavior (COM-B) model to examine correlates of university students’ physical activity behavior from three perspectives: educational contextual support, individual motivational processes, and external environmental opportunities. More specifically, SDT provides a theoretical lens for understanding why autonomy-supportive PE may be associated with students’ motivation for PA, whereas COM-B provides a complementary lens for understanding when such motivation may show a stronger association with actual physical activity. In this integrated framework, autonomy-supportive PE is viewed as relevant to the motivation component of COM-B because SDT suggests that autonomy-supportive contexts may support students’ experiences of autonomy, competence, and relatedness; competence-related experiences may also overlap conceptually with psychological capability. Environmental support corresponds most closely to the opportunity component of COM-B because facility access, interpersonal support, and informational resources may represent contextual conditions under which motivation is more strongly associated with PA. Although need satisfaction and capability were not directly measured, this alignment helps connect SDT’s motivational process with COM-B’s broader behavior framework. Accordingly, this study examines whether autonomy support in physical education is associated with university students’ physical activity, whether this association may be explained by motivation for physical activity, and whether environmental support moderates the relationship between motivation for physical activity and actual physical activity.

## 2 Literature review

### 2.1 The relationship between autonomy support in physical education and university students’ physical activity

As noted above, physical education (PE) is an important context for supporting students’ physical activity (10). From a public health perspective, physical education (PE) is one of the few routine educational settings that can repeatedly reach large numbers of young people through existing institutional structures (16). In physical education classes, physical education teachers, as important social agents in university students’ physical activity contexts, may provide autonomy support that serves as an important social factor associated with students’ exercise adherence (14, 18).

In physical education, autonomy support refers to an SDT-based teaching approach in which teachers acknowledge students’ perspectives and needs, provide meaningful choices, support self-directed decision-making, align instruction with students’ interests, and communicate the value of learning tasks (15, 19–21). In sport and exercise contexts, autonomy support has been associated with broadly beneficial motivational and behavioral outcomes (22, 23). More specifically, a meta-analysis of self-determination theory–based research in physical education found that teacher autonomy support was consistently associated with more adaptive student responses, including physical activity both within and beyond PE classes (24). In addition, empirical evidence from university students showed that autonomy support in PE was positively associated with physical activity (25). Based on these findings, we hypothesized that autonomy support in PE would be positively associated with university students’ physical activity.

### 2.2 The indirect association through motivation for physical activity

While previous research suggests that autonomy support in PE may be positively associated with university students’ physical activity, focusing only on the direct association may be insufficient to clarify possible explanatory pathways underlying this association. According to self-determination theory (SDT), motivation for physical activity may represent a theoretically relevant psychological pathway in the association between autonomy support in physical education and physical activity (14, 24, 26). Moreover, existing research has primarily focused on motivational and behavioral outcomes within physical education itself, such as motivation in PE classes (16) and participation in PE classes (15). In contrast, relatively limited research has examined whether autonomy support in university PE is associated with students’ motivation for physical activity beyond the classroom and whether this motivation is further associated with actual out-of-class physical activity. Therefore, it is necessary to further examine whether motivation for physical activity may help explain the association between autonomy support in PE and university students’ physical activity.

Autonomy support in PE may be positively associated with university students’ motivation for physical activity. According to self-determination theory, supportive behaviors may facilitate the internalization of motivation, and autonomy support in PE may be associated with stronger motivation among students (24). Autonomy support in PE may be associated with students’ motivation for physical activity through need-related and attitudinal processes. On the one hand, autonomy-supportive contexts are theorized to support individuals’ psychological needs in exercise contexts (26). On the other hand, autonomy support in PE may also be associated with more positive cognitions and attitudes toward physical activity among university students, which may in turn be related to stronger motivation for physical activity (27). Compared with support from peers and parents, autonomy support in PE (from PE teachers) has been found to show a stronger association with physical activity motivation (21). In addition, empirical studies have shown that autonomy support in PE is positively associated with out-of-class or leisure-time physical activity motivation (16). Taken together, these findings suggest that autonomy support in PE may be positively associated with motivation for physical activity.

Motivation for physical activity may be positively associated with university students’ physical activity. As an important psychological determinant of physical activity (28), motivation for physical activity is considered a key proximal factor associated with physical activity behavior (29). Specifically, individuals with higher motivation for physical activity may be more likely to devote greater time and effort to physical activity for reasons such as health promotion, appearance improvement, enjoyment, skill development, or social interaction (30). Accordingly, university students with stronger motivation for physical activity may also report higher levels of physical activity. Empirical studies have further shown that motivation for physical activity is positively associated with university students’ physical activity (28–32). Taken together, these findings suggest that motivation for physical activity may be positively associated with university students’ physical activity.

Taken together, prior theory and empirical evidence suggest that autonomy support in PE may be positively associated with motivation for physical activity, and that motivation for physical activity may in turn be positively associated with university students’ physical activity. Consistent with the trans-contextual model of motivation, autonomy support in PE may be associated with motivation beyond the classroom context, which may be further related to out-of-class physical activity (16, 33). However, relatively limited evidence has examined the potential spillover of autonomy-supportive PE from in-class experiences to out-of-class physical activity. Accordingly, the association between autonomy support in PE and physical activity may be indirectly linked through motivation for physical activity.

### 2.3 The conditional role of environmental support for physical activity

Although self-determination theory (SDT) has been effective in explaining university students’ inclination (including motivation and intention) toward physical activity, it is less adequate in predicting actual physical activity behavior. As demonstrated by Pan et al. (34), university students’ motivation for physical activity is not always strongly associated with actual behavior, suggesting that this motivation–behavior association may vary across conditions. Previous research has therefore called for the integration of multilevel theoretical perspectives to more comprehensively examine the interplay of individual, social, and environmental factors in predicting physical activity behavior (17). Against this background, it is important to further explore the conditions under which physical activity motivation is more strongly associated with actual behavior. According to the COM-B model, capability, opportunity, and motivation jointly contribute to physical activity behavior (35). These opportunity conditions may involve social influences as well as physical and environmental resources (11). In the university context, environmental conditions supportive of physical activity, such as conducive physical environments, positive interpersonal support, and accessible relevant information resources, may therefore serve as critical opportunity factors under which students’ motivation for physical activity may be more strongly associated with actual physical activity behavior.

However, existing research on university students’ physical activity has largely examined physical environmental factors (e.g., the availability of facilities) or social environmental factors (e.g., social support) separately (17), while relatively little attention has been paid to a more integrated environmental perspective. Moreover, in the digital era, the accessibility of physical activity-related information has also become an important factor associated with physical activity participation (9, 36–39). Therefore, the present study conceptualizes environmental support for physical activity as the extent to which an individual’s environment supports participation in physical activity through three dimensions: physical environmental support, social environmental support, and informational support. Specifically, physical environmental support includes factors such as the distance to sports facilities, facility standards, and venue conditions; social or interpersonal environmental support refers to the role-modeling and supportive influences of significant others; and informational support refers to the accessibility and ease of obtaining physical activity-related information (40).

Environmental support for physical activity may moderate the association between motivation for physical activity and physical activity. According to the COM-B model, motivation is not always strongly associated with behavior; rather, the occurrence of behavior also depends on whether individuals have sufficient opportunity conditions (41). Building on this view, West and Michie (42) further argued that opportunity is not only relevant to behavioral enactment itself, but may also be associated with the strength of the motivation–behavior association. First, Brown et al. (11) suggested that physical and social interpersonal environments provide more opportunities for physical activity behavior. Second, in university settings, guidance regarding the accessibility of environments and facilities, as well as informational resources such as the organization and promotion of physical activity events or sports activities, represent important ways through which university students become aware of and make use of opportunities for physical activity (39). Therefore, environmental support may provide more favorable opportunity conditions under which motivation for physical activity is more strongly associated with actual physical activity. However, previous studies have more often treated environmental support as a factor directly associated with physical activity, showing that the physical environment (37), the social interpersonal environment (8), and informational resources (36) are all closely related to physical activity among university students. By contrast, relatively limited research has further examined whether the positive association between motivation for physical activity and actual physical activity differs across levels of environmental support. Therefore, from the perspective of the COM-B model, the present study proposes that environmental support may represent a key condition under which motivation is more strongly associated with physical activity. Specifically, compared with low environmental support, the positive association between motivation for physical activity and physical activity may be stronger under conditions of high environmental support. Based on this, the present study hypothesized that environmental support moderates the association between motivation for physical activity and physical activity.

### 2.4 The current study

Although a growing body of research has examined autonomy support in physical education (PE) and its potential relevance to students’ physical activity behaviors, important questions remain insufficiently addressed. First, evidence is still limited regarding whether autonomy support in PE can spill over beyond the classroom, being associated with university students’ out-of-class motivation for physical activity and, in turn, with actual physical activity behavior. Second, it remains unclear whether environmental support for physical activity, as a key “opportunity” condition, is associated with a stronger motivation–behavior link.

To address these gaps, the present study integrates SDT and the COM-B model to examine university students’ physical activity from the perspectives of educational contextual support, motivational processes, and environmental opportunity conditions. Specifically, we aim to examine whether (1) autonomy support in PE is associated with physical activity behavior/volume, (2) motivation for physical activity mediates this association, and (3) environmental support for physical activity moderates the motivation–behavior link such that the association is stronger when environmental support is higher. Accordingly, we propose the following hypotheses:

*H1*: Autonomy support in PE is positively associated with physical activity.

*H2*: Motivation for PA mediates the association between autonomy support in PE and physical activity.

*H3*: Environmental support for PA positively moderates the association between motivation for PA and physical activity.

**Figure 1:**
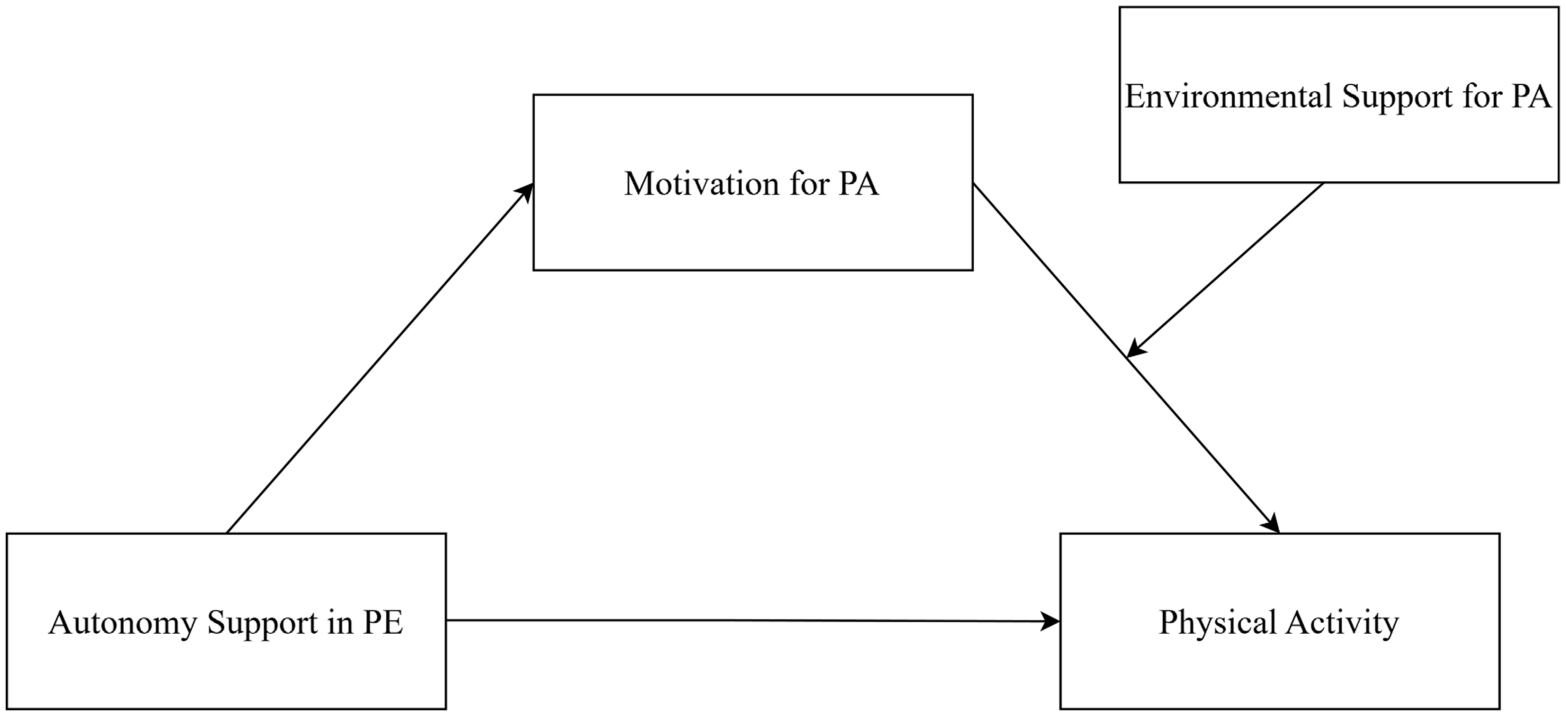
Hypothesized moderated mediation model.

The hypothesized moderated mediation model of the present study is shown in Figure 1. From a public health perspective, by jointly considering a modifiable educational factor (autonomy-supportive teaching in PE) and actionable environmental opportunity conditions (facility, social/interpersonal, and informational support), this study may help identify practical targets for supporting physical activity among university students and may provide evidence to inform campus-based health promotion policies and practices.

## 3 Method

### 3.1 Participants and data collection

A convenience sampling approach was used to collect data. The study questionnaire was developed and administered via the online survey platform Wenjuanxing^1^. The survey link was disseminated through physical education course–related student groups/class chat groups across five universities in three cities (Wuhan, Chongqing, and Foshan) to recruit undergraduate students who were enrolled in general physical education courses during the semester. Students who were physical education majors or professional athletes were excluded, as were those not enrolled in a general physical education course during the semester.

The introduction to the questionnaire informed participants that the study followed principles of anonymity and confidentiality. No personally identifiable information (e.g., name, home address, or national identification number) was collected. Data were used solely for academic research purposes. Participation was entirely voluntary, and participants could withdraw at any time without any adverse consequences. After reading and providing informed consent, participants proceeded to complete the questionnaire. All items were set as mandatory; therefore, no item-level missing data were present in the submitted responses. This study was reviewed and approved prior to data collection by the institutional ethics committee affiliated with the first author’s university on September 25, 2025 (approval number: IRB-202509079A). All procedures involving human participants were conducted in accordance with the ethical standards of the institutional review board and complied with the principles of the Declaration of Helsinki and its later amendments.

After data screening, for eligibility (i.e., current enrollment in a general physical education course), the final analytical sample comprised 1,024 undergraduate students, including 539 men (52.6%) and 485 women (47.4%). The mean age was 18.61 years (SD = 0.90).

### 3.2 Measure

#### 3.2.1 Perceived autonomy support in physical education

We adapted the Learning Climate Questionnaire [LCQ (43)] to the physical education context to assess the extent of students’ perceived autonomy support from their PE teacher(s). The scale contains 15 items rated on a 7-point Likert scale (1 = strongly disagree, 7 = strongly agree). A sample item is: “My PE teacher(s) tried to understand how I saw things before suggesting a new way to do something.” Item scores were averaged, with higher scores indicating greater perceived autonomy support in physical education. In the present sample, internal consistency was excellent (Cronbach’s *α* = 0.98).

#### 3.2.2 Motivation for physical activity

Motivation for physical activity was assessed using a 15-item Chinese version of the Motivation for Physical Activity Measure-Revised [MPAM-R (44)] adapted and shortened by Chen et al. (45). Items were rated on a 5-point Likert scale ranging from 1 (“not at all”) to 5 (“very strongly”), reflecting the strength of participants’ motivation for engaging in physical activity. A sample item is: “I want to maintain strength and live healthily.” Item scores were averaged, with higher scores indicating stronger motivation for physical activity. Internal consistency in the present sample was excellent (Cronbach’s *α* = 0.96).

#### 3.2.3 Environmental support for physical activity

Perceived environmental support for physical activity was assessed using an 11-item scale adapted from Liu et al. (40). The original instrument was developed in the context of table tennis; in the present study, item wording was generalized to refer to physical activity/exercise more broadly (e.g., replacing sport-specific terms with “physical activity” or “exercise”), while retaining the original meaning of each item. The scale covers three facets (facility environment support, interpersonal support, and informational support) and uses a 5-point Likert response format ranging from 1 (“strongly disagree”) to 5 (“strongly agree”). Sample items include: “It is convenient to travel from where I usually live, work, or study to exercise facilities,” “My close friends support me to exercise,” and “I can easily search for information related to physical activity.” In the present study, item scores were averaged to yield an overall score, with higher scores indicating greater perceived environmental support for physical activity. Internal consistency in the present sample was excellent (Cronbach’s *α* = 0.92).

#### 3.2.4 Physical activity

Physical activity was assessed using the Physical Activity Rating Scale–3 (PARS-3), a brief 3-item self-report instrument assessing exercise intensity, duration, and frequency, translated and revised for Chinese samples by Liang (46). The PARS-3 has been widely used to assess physical activity levels in Chinese student populations. A total physical activity score was computed as intensity × (duration − 1) × frequency, yielding a score ranging from 0 to 100, with higher scores indicating higher levels of physical activity. Because the PARS-3 score is a formula-based composite index rather than a reflective multi-item scale, internal consistency estimates are not strictly applicable.

#### 3.2.5 Physical literacy

Physical literacy was assessed using the simplified Chinese version of the Perceived Physical Literacy Instrument, as validated among Mainland Chinese undergraduates by Ma et al. (47). The scale contains 8 items rated on a 5-point Likert scale (1 = strongly disagree, 5 = strongly agree). A sample item is: “I possess adequate fundamental movement skills.” Item scores were averaged, with higher scores indicating higher perceived physical literacy, characterized by stronger motivation and confidence for movement and physical activity, greater perceived movement competence, and better perceived ability to interact effectively with the physical environment. In the present sample, internal consistency was excellent (Cronbach’s *α* = 0.91).

### 3.3 Statistical strategy

Descriptive statistics, reliability analyses, and bivariate correlations were computed using IBM SPSS Statistics 25.0. Given that all study variables were obtained via self-report, the possibility of common method bias (CMB) could not be ruled out. Therefore, Harman’s single-factor approach was used to assess potential CMB. First, a one-factor exploratory factor analysis was conducted in SPSS. Subsequently, a single-factor confirmatory factor analysis was performed in Mplus 8.3.

Hypotheses were tested using Hayes’ PROCESS macro (version 3.4) for SPSS. In line with Hayes (48), we used Model 14 to examine the hypothesized conditional process pattern. Given the cross-sectional design, the model was used to estimate associations and conditional indirect associations rather than to draw causal inferences about mediation. Because the study examined environmental correlates of university students’ physical activity, gender, age, and perceived physical literacy were included as covariates given their documented associations with physical activity in young adult populations (49). Controlling for these individual characteristics was intended to reduce potential confounding and improve the interpretability of the associations specified in the model. All continuous variables were standardized (*z*-scores) prior to analysis, whereas gender was treated as a categorical variable. Conditional indirect effects and the index of moderated mediation were estimated using bootstrap resampling (5,000 samples); indirect associations were considered statistically supported when the 95% bootstrap confidence interval did not include zero. To probe the interaction, simple slope analyses were conducted at low (−1 SD), mean, and high (+1 SD) levels of environmental support, and the Johnson–Neyman technique was used to identify regions of significance for the conditional effect of motivation on physical activity.

### 3.4 Common method bias

Given that all variables were assessed via self-report questionnaires, common method bias (CMB) was considered a potential source of measurement artifact. Following Harman’s single-factor approach, we first conducted an exploratory factor analysis in SPSS with all self-report scale items entered simultaneously. Using principal axis factoring with an unrotated solution, the first factor explained 45.66% of the variance [below the commonly used 50% criterion (50)], suggesting that a single factor did not account for the majority of the covariance among the measures. As a supplementary check, we further tested a single-factor confirmatory factor analysis in Mplus 8.3, in which all self-report indicator items were specified to load on one common latent factor. The single-factor model showed poor fit to the data (CFI = 0.56, TLI = 0.54, RMSEA = 0.10, SRMR = 0.13), providing additional evidence that CMB was unlikely to represent a dominant source of shared variance among the study variables (51). Taken together, these results do not indicate serious common method bias in the present study.

## 4 Results

### 4.1 Descriptive statistics and correlational analyses

Descriptive statistics and Pearson correlations are presented in Table 1. Autonomy support in PE was positively correlated with motivation for PA (*r* = 0.55, *p* < 0.001) and physical activity (*r* = 0.27, *p* < 0.001). Motivation for PA was positively correlated with physical activity (*r* = 0.33, *p* < 0.001). Environmental support for PA was positively correlated with physical activity (*r* = 0.37, *p* < 0.001). All variance inflation factors (VIFs) were well below 5, suggesting no evidence of problematic multicollinearity.

**Table 1:** Descriptive statistics and correlations.

Variable	*M*	*SD*	1	2	3	4	5	6	VIF
1. Gender	–	–	–						1.04
2. Age	18.61	0.90	0.10^**^	–					1.01
3. Physical literacy	3.77	0.71	0.09^**^	0.05	–				2.38
4. Autonomy support in PE	5.43	1.13	0.03	0.04	0.61^***^	–			2.06
5. Environmental support for PA	3.73	0.72	0.15^***^	0.03	0.73^***^	0.67^***^	–		2.63
6. Motivation for PA	3.76	0.88	0.06^*^	0.05	0.55^***^	0.55^***^	0.52^***^	–	1.61
7. Physical activity	19.99	19.43	0.27^***^	0.09^**^	0.38^***^	0.28^***^	0.37^***^	0.33^***^	–

*N* = 1,024. Gender was coded as 0 = female, 1 = male. Autonomy support in PE = perceived autonomy support from PE teachers; Environmental support for PA = perceived environmental support for physical activity; Motivation for PA = motivation for physical activity; Physical activity = PARS score. Values on the off-diagonal are Pearson correlation coefficients based on mean scores. VIF = Variance Inflation Factor. ^*^p < 0.05, ^**^p < 0.01, ^***^p < 0.001. Abbreviations and significance codes are used consistently throughout.

### 4.2 Moderated mediation model

Results of the moderated mediation model are summarized in Tables 2, 3. As shown in Table 2, autonomy support in PE was positively associated with motivation for PA (*β* = 0.335, *p* < 0.001), but the direct association between autonomy support in PE and physical activity was not statistically supported (*β* = 0.001, *p* = 0.999); motivation for PA was positively associated with physical activity (*β* = 0.157, *p* < 0.001), and this association was moderated by environmental support for PA (*β* = 0.067, *p* = 0.002). Thus, although autonomy support in PE was positively correlated with physical activity at the bivariate level, H1 was not supported in the full conditional process model after motivation for PA, environmental support for PA, and their interaction were taken into account.

**Table 2:** Regression results for the moderated mediation model.

Outcome variables	Independent variables	*R*	*R^2^*	*F*	*β*	*t*	*p*	95% CI
Motivation for PA		0.612	0.375	152.556^***^				
	Gender				0.043	0.810	0.396	[−0.056, 0.141]
	Age				0.020	0.810	0.418	[−0.029, 0.069]
	Autonomy support in PE				0.335	10.735	<0.001	[0.274, 0.396]
	Physical literacy				0.344	10.986	<0.001	[0.283, 0.406]
Physical activity		0.482	0.232	43.830^***^				
	Gender				0.423	7.485	<0.001	[0.312, 0.534]
	Age				0.046	1.645	0.100	[−0.009, 0.100]
	Autonomy support in PE				0.001	0.001	0.999	[−0.076, 0.076]
	Motivation for PA				0.157	4.469	<0.001	[0.088, 0.226]
	Environmental support for PA				0.109	2.441	0.015	[0.021, 0.197]
	Environmental support × motivation				0.067	3.063	0.002	[0.024, 0.109]
	Physical literacy				0.198	4.666	<0.001	[0.115, 0.281]

Continuous variables were *z*-standardized prior to analysis; therefore, the reported coefficients can be interpreted as standardized effects (*β*). Gender was coded as 0 = female, 1 = male. 95% CIs are based on standard errors. *p*-values are two-tailed. *** indicates *p* < 0.001.

**Table 3:** Conditional effects and conditional indirect effects across levels of environmental support for PA.

Effect	Moderator level	*β*	SE	LLCI	ULCI	*t*	*p*
Conditional effect of motivation for PA on physical activity (M → Y)	Low (−1 SD)	0.091	0.039	0.014	0.167	2.325	0.020
	Mean	0.157	0.035	0.088	0.226	4.469	<0.001
	High (+1 SD)	0.224	0.044	0.138	0.310	5.132	<0.001
	Moderator level	Effect	BootSE	Boot LLCI	Boot ULCI		
Conditional indirect effects (X → M → Y)	Low (−1 SD)	0.030	0.014	0.006	0.059		
	Mean	0.053	0.013	0.029	0.079		
	High (+1 SD)	0.075	0.018	0.040	0.111		

Moderator = environmental support for PA. Bootstrap confidence intervals are based on 5,000 resamples.

**Figure 2:**
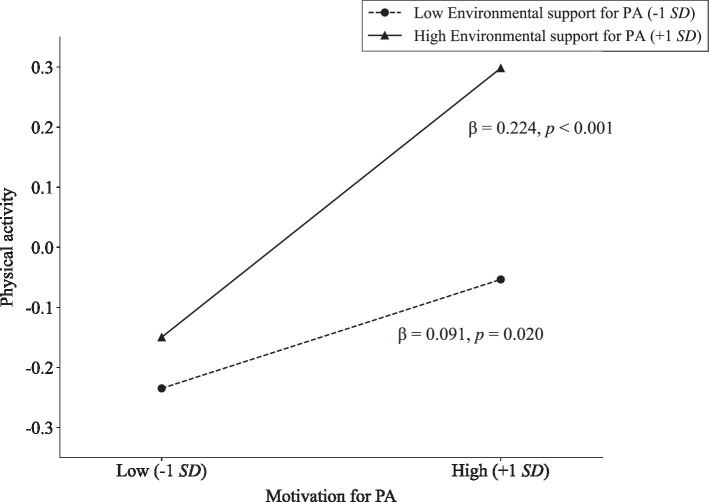
Relationship between motivation for PA and physical activity at low and high levels of environmental support for PA.

To further interpret the significant interaction, simple slope analyses were conducted at low (−1 SD) and high (+1 SD) levels of environmental support for PA. As shown in Table 3 and Figure 2, motivation for PA was positively associated with physical activity under low environmental support (*β* = 0.091, *p* = 0.020), and this positive association was stronger under high environmental support (*β* = 0.224, *p* < 0.001).

**Figure 3:**
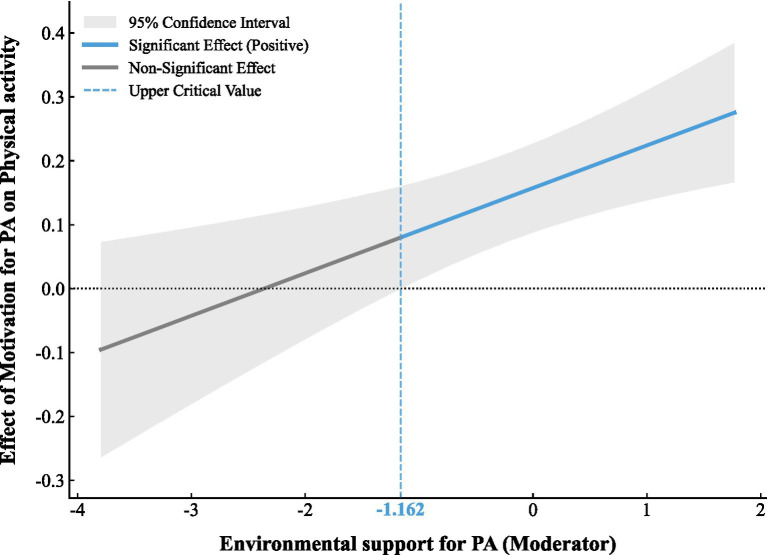
Johnson-Neyman analysis.

To complement the simple slope analyses (which probe the interaction at selected values of the moderator), we applied the Johnson–Neyman technique to identify the range of environmental support over which the conditional association between motivation and physical activity is statistically supported across the full continuum of the moderator. The Johnson–Neyman analysis (Figure 3) showed that when environmental support for PA was ≤−1.16 SD, the conditional effect of motivation for PA on physical activity was not statistically supported (8.40% of the sample); when environmental support for PA was >−1.16 SD, the conditional effect was statistically supported (91.60% of the sample).

Moreover, conditional indirect effects of autonomy support in PE on physical activity via motivation for PA are presented in Table 3. The indirect association was statistically supported at low (−1 SD; indirect effect = 0.030, BootSE = 0.014, 95% bootstrap CI [0.006, 0.059]) and high (+1 SD; indirect effect = 0.075, BootSE = 0.018, 95% bootstrap CI [0.040, 0.111]) levels of environmental support for PA, with a larger indirect effect observed under higher environmental support. Consistently, the index of moderated mediation was statistically different from zero (index = 0.022, BootSE = 0.009, 95% bootstrap CI [0.003, 0.040]). Overall, these results indicate that the indirect association between autonomy support in PE and physical activity through motivation for PA varied by levels of environmental support for PA, such that the estimated indirect association was stronger at higher levels of environmental support. Therefore, in the full conditional process model, H1 was not supported, whereas H2 and H3 were statistically supported. These findings suggest that the association between autonomy support in PE and physical activity was more clearly reflected as an indirect association through motivation for PA, conditional on environmental support for PA.

## 5 Discussion

Drawing on self-determination theory and the COM-B model, this study examined a theoretically informed conditional process model of associations among perceived autonomy support in PE, motivation for PA, environmental support for PA, and physical activity among Chinese university students. Overall, the findings suggest that autonomy support in PE was positively associated with motivation for PA, which in turn was positively associated with physical activity. In addition, environmental support was associated with a stronger association between motivation and physical activity. From a public health perspective, these findings underscore the value of considering both educational and environmental influences on physical activity in university settings.

### 5.1 The indirect association through motivation for PA

A key finding of the present study is that motivation for PA may help explain the association between perceived autonomy support in PE and physical activity among Chinese university students. Notably, however, the direct association between perceived autonomy support in PE and physical activity was not statistically significant after motivation for PA, environmental support, and their interaction were taken into account. This finding means that H1 was not supported in the full model, although the bivariate correlation between autonomy support in PE and physical activity was positive. As suggested by Bi et al. (29), contextual variables may function as more distal correlates of physical activity and may need to operate through more proximal factors, such as motivation, to be associated with behavior. In this regard, the present pattern suggests that autonomy support in PE may function as a relatively distal correlate of physical activity, whereas motivation for PA may represent a more proximal correlate within the association between supportive educational experiences and students’ physical activity behavior. Accordingly, the findings highlight motivation as a possible explanatory pathway in the association between autonomy-supportive PE and physical activity.

This interpretation is broadly consistent with self-determination theory, which suggests that autonomy-supportive contexts are theorized to facilitate the internalization of motivation and that more self-determined motivation may in turn be associated with physical activity (14, 24, 26). First, autonomy support in PE was positively associated with motivation for PA, which may be understood from at least two perspectives. On the one hand, autonomy-supportive teaching may be associated with conditions that support students’ basic psychological needs in movement-related contexts, which may in turn be associated with stronger motivation for PA (26). On the other hand, autonomy support in PE may also be associated with more positive cognitions and attitudes toward physical activity, which may be related to students’ greater willingness to value and pursue physical activity beyond the classroom (27). Second, motivation for PA was positively associated with physical activity. As motivation for PA is regarded as a more proximal correlate of physical activity behavior (29), stronger motivation may help explain higher levels of physical activity among university students (28, 30). These patterns are consistent with an SDT-based interpretation of the indirect association.

The present study also adds to the literature from a trans-contextual perspective. Previous research has focused primarily on motivational and behavioral outcomes within PE itself, such as motivation in PE classes and participation or engagement in PE activities (15, 16). By contrast, relatively limited attention has been paid to whether autonomy-supportive experiences in university PE are associated with students’ motivation for PA beyond the classroom and whether such motivation is further associated with actual out-of-class physical activity. The current findings extend this line of work by suggesting that autonomy-supportive teaching in PE may have relevance not only for students’ in-class experiences, but also for their broader motivation for PA and physical activity in everyday life. This interpretation is in line with the trans-contextual model of motivation, which proposes that supportive experiences in educational settings may be associated with motivation and behavior beyond the original context (16, 33). In this sense, the findings underscore the potential theoretical value of viewing PE not only as a curriculum-based instructional setting, but also as a context in which supportive pedagogical practices may be relevant to extracurricular health behavior.

### 5.2 The conditional role of environmental support for PA

A further key finding of the present study is that environmental support for PA moderated the association between motivation for PA and physical activity. This suggests that the indirect association through motivation for PA varied by the level of environmental support. More specifically, when environmental support was higher, motivation for PA showed a stronger positive association with physical activity, and the indirect association between perceived autonomy support in PE and physical activity through motivation was more evident. By contrast, when environmental support was lower, the indirect association through motivation was weaker under lower environmental support, suggesting that motivation may show a weaker association with actual physical activity under less supportive conditions.

This pattern can be understood from the perspective of the COM-B model, which emphasizes that behavior depends not only on motivation but also on opportunity conditions (35, 41), and that opportunity is relevant not only to behavioral enactment itself, but also to the strength of the motivation–behavior association (42). In other words, even when university students are motivated to be physically active, such motivation may not always be strongly associated with actual behavior (34), and this association may be stronger when sufficient environmental opportunities are available. In previous research, different dimensions of environmental support have often been treated as direct contextual correlates or predictors of physical activity (8, 36, 37). The present study extends this perspective by suggesting that environmental support may also function as an opportunity condition under which motivation is more strongly associated with behavior. From this perspective, environmental support functions as a boundary condition for the motivational process: it may be associated with variation in the strength of the motivation–behavior link.

This interpretation may also be further illustrated by considering the possible roles of different dimensions of environmental support. Physical environmental or facility support may be associated with greater opportunities for physical activity by improving access to spaces, equipment, and infrastructure, thereby reducing the practical effort required to initiate and maintain physical activity (11). Social or interpersonal support may be associated with stronger behavioral enactment by providing encouragement, companionship, role modeling, and normative reinforcement, which is consistent with prior work highlighting the role of supportive social environments in physical activity opportunities (11). Informational support may further be associated with perceived opportunity by helping students recognize available resources, understand how to participate, and make use of campus-based activity options (39). Taken together, these dimensions suggest that environmental support may function not only as a background correlate of physical activity, but also as an opportunity condition under which motivation is more likely to be associated with physical activity in everyday university life.

Taken together, these findings highlight the value of integrating SDT and COM-B in understanding PA among university students. SDT helps explain why autonomy-supportive PE may be associated with motivation for PA, whereas COM-B helps explain why the motivation–PA association may vary depending on opportunity conditions. This interpretation is consistent with the “seed-and-soil” logic described by Walton and Yeager (52): autonomy-supportive PE may be understood as part of the motivational “seed,” whereas environmental support may represent the contextual “soil” under which this motivation is more likely to be associated with actual PA. From a public health perspective, PA in university settings may therefore be better understood in relation to both motivational formation and contextual enablement.

### 5.3 Practical implications

Although the observed associations were statistically supported, their magnitudes should be interpreted with appropriate caution. Given that university students’ physical activity is influenced by multiple individual, interpersonal, institutional, and environmental factors, the modest size of the observed associations is not unexpected. From a public health perspective, these findings may still have practical relevance because they concern modifiable educational and environmental factors that can be addressed through routine PE courses and campus-level health promotion strategies.

At the educational level, the present findings suggest that PE may serve as a practical and scalable context for supporting physical activity among university students. Because autonomy-supportive PE was associated with stronger motivation for PA, universities may benefit from strengthening PE teachers’ autonomy-supportive practices through professional development, such as offering meaningful choice, acknowledging students’ perspectives, using non-controlling language, and explicitly communicating the value of physical activity (15, 19–21). In Chinese university general PE courses, this could include allowing students to choose among different activity modules when feasible, adapting tasks to students with different skill levels, explaining the health value of required exercises, and encouraging students to set feasible out-of-class PA goals. Recent intervention research further suggests that autonomy-supportive teaching is not fixed, but can be enhanced through targeted teacher training, with potential benefits for students’ motivation (53). In addition, PE courses may be better positioned to support physical activity when they extend beyond in-class participation and help students connect class experiences with out-of-class action. For example, school-based programs that incorporate autonomy support, positive reinforcement, goal setting, and interest cultivation may support the connection between motivation and physical activity beyond the classroom (54).

At the campus environment level, the present findings suggest that universities may further strengthen opportunity conditions under which students’ motivation is more likely to be associated with physical activity. First, facility support may be strengthened by improving the accessibility, convenience, and usability of exercise spaces, equipment, and campus infrastructure, which may make physically active choices easier to embed in students’ daily routines and provide more opportunities for physical activity (11). In practical terms, universities could extend the opening hours of sports facilities, improve access to fields and courts outside class time, provide transparent venue-booking systems, and ensure that students in different dormitory areas can conveniently access exercise spaces. Second, social or interpersonal support may also be cultivated. As Gao et al. (55) showed in their systematic review, social capital—including support from friends and stronger social networks—was positively associated with higher physical activity among university students. This suggests that social or interpersonal support may be further strengthened by creating peer participation opportunities, partnership mechanisms, group-based programs, and visible norms that encourage active lifestyles. In Chinese university settings, class groups, dormitory networks, student associations, and campus sport clubs may serve as useful channels for organizing peer-based PA activities and fostering supportive exercise norms. Third, informational support may also be made more actionable through timely and centralized communication about facilities, activity schedules, campus programs, and practical ways to participate. Consistent with evidence that university interventions frequently use social media, mobile applications, web-based tools, and online messaging (56), such information could be delivered through campus apps, WeChat public accounts, course chat groups, or student affairs platforms, including facility availability, beginner-friendly exercise options, and reminders about campus sport events. Taken together, these implications suggest that when facility, social, and informational supports work in coordination, stronger campus environmental support may be associated with a stronger link between students’ motivation for PA and actual physical activity behavior.

More broadly, the present findings support an integrated approach to campus physical activity promotion that considers both motivational formation and environmental opportunity, consistent with evidence that university-based PA interventions often combine multiple strategies rather than relying on a single component (56). For example, PE departments, student affairs offices, campus facility managers, and student sport organizations could coordinate course-based PA guidance, venue access, peer activities, and digital communication so that motivational and environmental supports are aligned. Such coordination may help create campus environments in which students’ motivation is more strongly connected with physically active behavior in everyday life.

### 5.4 Limitation and future research

Nevertheless, several limitations of the present study should be acknowledged. First, the study employed a cross-sectional design. Although the relationships among variables were explored and interpreted based on relevant theories and prior empirical evidence, the nature of the design does not allow causal inference, clear determination of the temporal ordering among variables, or causal interpretation of the indirect association. Specifically, the present data cannot determine whether perceived autonomy support in PE preceded motivation for PA, or whether motivation for PA preceded subsequent physical activity. Relatedly, alternative explanations cannot be ruled out. For example, students who are already more physically active may report stronger motivation for PA or perceive PE teachers and campus environments more positively, and unmeasured variables such as prior sport experience, personality traits, course type, or campus sport culture may partly account for the observed associations. Therefore, the observed indirect association should be interpreted as a statistically estimated and theoretically guided pattern rather than evidence of a confirmed mediation mechanism. Future research is encouraged to adopt longitudinal, cross-lagged, experimental, or intervention-based designs to examine whether changes in autonomy-supportive PE are followed by changes in motivation for PA and whether subsequent changes in motivation are followed by changes in physical activity behavior. Second, physical activity data in the present study were primarily obtained through self-report measures, which may be subject to recall bias and social desirability bias. As a result, some discrepancy may exist between the reported data and individuals’ actual levels of physical activity. More broadly, because all study variables were collected using self-report questionnaires, common method bias may also be a concern. Although Harman’s single-factor test and a single-factor CFA were conducted to assess potential common method bias, these procedures have limitations and cannot fully rule out common method variance. Future studies may therefore incorporate objective indicators of physical activity, such as wearable activity trackers, accelerometers, or step-count records, combine subjective and objective measures (57, 58), and use stronger remedies for common method bias, such as temporal separation, multi-source data, or marker variables. Third, the sample was drawn from Chinese university students, and the representativeness of the sample may have been relatively limited. Accordingly, caution is needed when generalizing the findings to other populations or educational contexts. Future research may further expand the sample size, improve sampling representativeness, or extend the research to other age groups and educational stages in order to test the robustness and broader applicability of the present findings. Furthermore, although the present study primarily examined how motivation for PA is associated with educational and environmental factors, and under what conditions such motivation may show a stronger association with physical activity behavior, individuals with different characteristics may still show different patterns of association and behavioral outcomes under the same environmental conditions (11, 17). Therefore, future research may further investigate potential heterogeneity across different subgroups, such as gender or physical literacy, so as to deepen understanding of possible explanatory pathways related to physical activity behavior. Finally, to test a parsimonious and theoretically integrated model, the present study used overall scores for several key constructs. This approach may have obscured meaningful differences among subdimensions. Specifically, motivation for PA was not separated into autonomous motivation, controlled motivation, and amotivation; environmental support was not examined separately in terms of facility, interpersonal, and informational support; and perceived autonomy support in PE was assessed as an overall construct, although recent research suggests that autonomy support may also be multidimensional (59). Future studies could examine whether different forms of autonomy support, types of motivation, and dimensions of environmental support show distinct associations with physical activity.

## 6 Conclusion

This study suggests that perceived autonomy support in PE may be positively associated with university students’ motivation for PA, and that motivation for PA may in turn be positively associated with physical activity. In addition, the association between motivation for PA and physical activity appears to be stronger under more supportive environmental conditions. These findings further suggest that autonomy-supportive experiences in PE may be associated with students’ motivation for physical activity beyond the classroom context, while supportive environments may help explain when such motivation is more strongly associated with out-of-class physical activity. Overall, the present findings underscore the importance of attending to both autonomy-supportive teaching practices and supportive environmental opportunities in campus-based physical activity promotion. From a public health perspective, efforts to support physical activity among university students may benefit from considering both motivational formation and environmental enablement.

## Data Availability

The datasets presented in this study can be found in online repositories. The names of the repository/repositories and accession number(s) can be found in the article/Supplementary material.

## Footnotes

1
https://www.wjx.cn/
