# Supplementary material for: Autonomy support in physical education and university students’ physical activity: indirect and conditional associations through motivation and environmental support
Source: Front Public Health. 2026 May 20;14:1854693. doi: 10.3389/fpubh.2026.1854693 (PMC13230099; doi:10.3389/fpubh.2026.1854693)
Supplement: Supplementary file 1 [file Supplementary_File_1.docx]

**Appendix. Measurement Scales**

Note. All variables in the present study were measured using Chinese-language questionnaires. For perceived autonomy support in PE, motivation for physical activity, and perceived physical literacy, the English wording is presented to show the corresponding original English items or study versions for reference. For environmental support for physical activity and physical activity level, the English wording is translated from the Chinese versions and is provided for reference only.

**Perceived Autonomy Support in PE**

*Response format: 1 = strongly disagree; 7 = strongly agree*

| **Code** | **Item** |
| --- | --- |
| AS1 | I feel that my PE teacher provides me with choices and options. |
| AS2 | I feel understood by my PE teacher. |
| AS3 | I am able to be open with my PE teacher during class. |
| AS4 | My PE teacher conveys confidence in my ability to do well. |
| AS5 | I feel that my performance is recognized by my PE teacher. |
| AS6 | My PE teacher makes sure that I really understand my exercise goals and what I need to do. |
| AS7 | My PE teacher encourages me to ask questions. |
| AS8 | I feel a lot of trust in my PE teacher. |
| AS9 | My PE teacher answers my questions fully and carefully. |
| AS10 | My PE teacher listens to how I would like to do things. |
| AS11 | My PE teacher handles people's emotions very well. |
| AS12 | I feel that my PE teacher cares about me as a person. |
| AS13 | I feel comfortable with the way my PE teacher talks to me. |
| AS14 | My PE teacher tries to understand how I see things before suggesting a new way to do things. |
| AS15 | I feel able to share my feelings with my PE teacher. |

**Motivation for Physical Activity**

*Response format: 1 = not at all; 2 = a little; 3 = moderately; 4 = strongly; 5 = very strongly*

| **Code** | **Item** |
| --- | --- |
| EM1 | Because I want to be physically fit. |
| EM2 | Because I want to control my body weight. |
| EM3 | Because I want to participate in enjoyable recreational activities. |
| EM4 | Because I want to obtain new exercise skills. |
| EM5 | Because I want to meet new friends. |
| EM6 | Because I want to maintain my physical and mental well-being. |
| EM7 | Because I want to maintain or improve my body shape. |
| EM8 | Because I want to maintain a positive mood. |
| EM9 | Because I want to improve my existing exercise skills. |
| EM10 | Because I want to strengthen my friendship and emotional bonds with friends. |
| EM11 | Because I want to lead a healthy lifestyle. |
| EM12 | Because I want to be more physically attractive. |
| EM13 | Because I want to enjoy a happy life. |
| EM14 | Because I want to maintain my current exercise skill level. |
| EM15 | Because I want to maintain good social relationships. |

**Environmental Support for Physical Activity**

*Response format: 1 = strongly disagree; 5 = strongly agree*

| **Domain** | **Code** | **Item** |
| --- | --- | --- |
| Facility support | ES1 | Transportation from where I usually live, work, or study to sports or exercise venues is relatively convenient. |
|  | ES2 | The facilities at the places where I usually exercise generally meet my needs. |
|  | ES3 | I am generally satisfied with the environment of the places where I usually exercise. |
|  | ES4 | When I want to exercise, I can usually find an ideal place without much difficulty. |
| Interpersonal support | ES5 | When I need it, there are people who can give me specific guidance and suggestions about exercise. |
|  | ES6 | I often have someone to exercise with. |
|  | ES7 | My friends exercise regularly. |
|  | ES8 | My friends support my participation in exercise. |
| Informational support | ES9 | I have favorite sports or exercise stars or role models. |
|  | ES10 | I can relatively easily find information related to exercise. |
|  | ES11 | I often browse or consult exercise-related information. |

**Physical Activity Rating Scale-3 (PARS-3)**

*Instruction: The following three questions assess your physical activity during the past month. For each question, please select the one option that best describes your situation.*

**1. What was the intensity of your physical activity?**

1. Light exercise (e.g., walking, radio calisthenics, casual racket games)

2. Low-intensity, not very strenuous exercise (e.g., recreational volleyball, table tennis, jogging, Tai Chi)

3. Moderate-intensity, relatively vigorous and sustained exercise (e.g., cycling, running, table tennis)

4. High-intensity exercise causing rapid breathing and heavy sweating, but not sustained for long (e.g., badminton, basketball, volleyball, football/soccer)

5. Sustained high-intensity exercise causing rapid breathing and heavy sweating (e.g., long-distance running, full aerobic exercise routines, swimming)

**2. How long did you usually engage in physical activity each time?**

1. Less than 10 minutes

2. 11-20 minutes

3. 21-30 minutes

4. 31-59 minutes

5. 60 minutes or more

**3. How often did you engage in physical activity during the past month?**

1. Less than once a month

2. Two to three times a month

3. One to two times a week

4. Three to five times a week

5. About once a day

Scoring: Physical activity score = intensity × (duration - 1) × frequency, ranging from 0 to 100.

**Perceived Physical Literacy**

*Response format: 1 = strongly disagree; 5 = strongly agree*

| **Code** | **Item** |
| --- | --- |
| PL1 | I possess adequate fundamental movement skills. |
| PL2 | I am physically fit in accordance with my age. |
| PL3 | I am able to apply learned motor skills to other physical activities. |
| PL4 | I appreciate myself or others doing sports. |
| PL5 | I have strong communication skills. |
| PL6 | I have strong social skills. |
| PL7 | I am aware of the health benefits of sports and exercise. |
| PL8 | I am eager to know current trends in sports and exercise. |

**Availability of data and materials**

The de-identified data used in this study have been uploaded to the Open Science Framework (OSF) and are available via the following view-only link: <https://osf.io/963bq/overview?view_only=08ae0fd79a6f4ff28383dd8615f954a6>
